# Supplementary material for: Mechanisms governing the pioneering and redistribution capabilities of the non-classical pioneer PU.1
Source: Nat Commun. 2020 Jan 21;11:402. doi: 10.1038/s41467-019-13960-2 (PMC6972792; doi:10.1038/s41467-019-13960-2)
Supplement: Supplementary file 7 — Source data [file 41467_2019_13960_MOESM7_ESM.zip › Source_Data/FigureS2/FigureS2B_MotifScanOutput/KmeansCluster11/homerResults/motif5.similar.html]

motif5

## Information for motif5

T
A
C
G
A
G
C
T
C
A
T
G
T
G
A
C
A
G
T
C
G
T
A
C
A
G
T
C
A
G
T
C
G
A
C
T
C
T
G
A
A
T
C
G
C
G
A
T
A
C
T
G
T
A
C
G
  
Reverse Opposite:  

A
T
G
C
A
G
T
C
G
C
T
A
T
A
G
C
G
A
C
T
C
T
G
A
C
T
A
G
C
T
A
G
C
A
T
G
C
T
A
G
A
C
T
G
G
T
A
C
C
T
G
A
A
T
G
C
  

|  |  |
| --- | --- |
| p-value: | 1e-92 |
| log p-value: | -2.131e+02 |
| Information Content per bp: | 1.688 |
| Number of Target Sequences with motif | 166.0 |
| Percentage of Target Sequences with motif | 5.11% |
| Number of Background Sequences with motif | 207.9 |
| Percentage of Background Sequences with motif | 0.46% |
| Average Position of motif in Targets | 89.6 +/- 53.4bp |
| Average Position of motif in Background | 99.3 +/- 63.6bp |
| Strand Bias (log2 ratio + to - strand density) | -0.3 |
| Multiplicity (# of sites on avg that occur together) | 1.00 |
| Motif File: | file (matrix) reverse opposite |

### Similar de novo motifs found

|  |  |  |  |  |  |  |  |
| --- | --- | --- | --- | --- | --- | --- | --- |
| Rank | Match Score | Redundant Motif | P-value | log P-value | % of Targets | % of Background | Motif file |
| 1 | 0.965 | G T A C A G T C C G T A T A G C A G T C C G T A T A C G T C A G C A T G C A T G A C T G G A T C C T G A | 1e-83 | -191.899137 | 4.28% | 0.33% | motif file (matrix) |
| 2 | 0.948 | G A T C A G T C C T G A T A G C G A T C C G T A C A T G C T A G A C T G C T A G A C T G G T A C | 1e-72 | -167.265014 | 5.14% | 0.70% | motif file (matrix) |
| 3 | 0.795 | G T C A T A G C G A C T C T G A A C T G C T G A C T A G C T A G A C T G T G A C T C A G | 1e-46 | -107.267318 | 3.29% | 0.45% | motif file (matrix) |
| 4 | 0.815 | T C G A T A G C G A C T C G T A A C T G C T G A A C T G C A T G A T C G G T A C | 1e-42 | -97.430807 | 3.76% | 0.69% | motif file (matrix) |
| 5 | 0.764 | T C G A A T G C A G C T G T C A C T A G T C G A A C T G C T A G C A T G | 1e-23 | -53.023680 | 4.19% | 1.47% | motif file (matrix) |
| 6 | 0.842 | A C T G G T A C A G T C C G T A A G T C A G T C G C A T T C G A A T C G C G A T C T A G | 1e-21 | -49.688242 | 1.69% | 0.27% | motif file (matrix) |
